# Mentalizing in individuals with state and trait risk for psychosis: a systematic review

**DOI:** 10.3389/fpsyt.2023.1214385

**Published:** 2023-10-17

**Authors:** Francesca De Salve, Chiara Rossi, Osmano Oasi

**Affiliations:** Department of Psychology, Catholic University of Sacred Heart, Milan, Italy

**Keywords:** At-Risk Mental States, schizotypy, CHR, UHR, mentalizing, metacognition, Theory of Mind (ToM)

## Abstract

**Background:**

Mentalization is an umbrella concept defined as the ability to interpret one’s and others’ mental states. Previous studies have hypothesized that mentalization may be a crucial resilience factor that significantly moderates the likelihood of developing psychotic disorders in individuals with both state and trait risk factors for the illness.

**Purpose:**

The study reviews the role of mentalizing abilities (e.g., reflective functioning, Theory of Mind (ToM), and metacognition) in young adults with At-Risk Mental States (ARMS) and schizotypal traits. Specifically, the objective is to include articles that (a) evaluate the links between low mentalizing and both state (ARMS/CHR) and trait (schizotypy) risk for psychosis (b) compare the differences in mentalizing abilities between individuals with ARMS, schizotypy, full-blown psychosis, and healthy controls.

**Method:**

Electronic databases (PsycINFO, PubMed, Scopus, and Google Scholar) were used to search for articles, while Rayyan was employed to facilitate the screening and selection of studies. Eligible studies are original English-language; peer-reviewed research articles on populations that met validated risk diagnostic criteria for psychosis, ARMS, and healthy controls; empirical studies evaluating the association or differences between psychotic risk and mentalizing abilities. Non-English language studies, the ones not considering state or trait risk for psychosis, and qualitative studies were excluded. After the application of the PRISMA checklist and the inclusion and exclusion criteria previously mentioned, 10 articles were extracted. The systematic review has been registered on Prospero (CRD42023397594).

**Results:**

Low levels of reflective functioning and metacognition may predict a transition to psychosis. In addition, reflective functioning and metacognitive impairments are associated with attenuated psychotic symptoms in both state risk groups and in non-clinical individuals with schizotypal traits. Concerning ToM tasks, mixed results emerged.

**Conclusion:**

The results obtained from the review suggest that the application of strategies to attenuate maladaptive metacognitive beliefs and low mentalization may be equally effective in improving psychotic symptoms. The assessment of mentalization and metacognition could potentially provide additional prognostic value over factors predisposing to psychosis. Good mentalization and metacognition functioning should be considered as protective factors able to minimize the transition to psychosis.

## 1. Introduction

Mentalizing refers to the capacity to understand ourselves and others’ behavior in terms of intentional mental states – i.e., feelings, desires, wishes, attitudes, and goals (1). It is a complex construct encompassing the capacity to deduce cognitive and emotional states pertaining to oneself and others. This underscores its dynamic character in relation to diverse contexts of interpersonal interactions (2). Given its complexity, in order to facilitate its measurement within studies, mentalizing has been operationalized through the introduction of the reflective function construct, which is often used synonymously with mentalization. Reflective function (RF) captures all the different facets of mentalizing, including mental state understanding for both cognitions and affects in oneself and others (3). Mentalization can be conceived as an umbrella term, covering related constructs from social cognition research including Theory of Mind (ToM) and metacognition (4, 5). ToM is the ability to make inferences about others’ thoughts and intentions. The term refers to the cognitive ability to attribute mental states to others and understand the link between mental states and actions (6). Metacognition has been primarily defined as the ability to “think about thinking” (7). It involves introspection of one’s own behavior, whereas ToM involves perceiving the mental states driving others’ behavior. It is unclear to what extent mentalization, ToM, and metacognition are independent mechanisms with distinct abilities that relate to different outcomes, or whether they share a common architecture that allows them to follow similar developmental paths and provide similar inputs (8). Nevertheless, ToM and metacognition may overlap mentalization, respectively, for the component directed toward others and for the cognitive component concerning awareness of thought. The shared identity of mentalization, metacognition, and Theory of Mind can be captured by the concept of Higher-Order Cognition (HOC) (9). HOC processes stem from hierarchical networks of information processing that allow for abstraction. They involve self-awareness and awareness of oneself in relation to others and the world. In the context of psychotic or first-episode psychotic patients, the role of HOC functions has been investigated in numerous studies (10–13). These studies have explored the significance of mentalizing abilities across the continuum of psychosis, leading to the hypothesis that enhancing HOC could assist individuals in reorganizing their cognitive processes, resulting in more flexible and adaptive models of reality testing (9, 14).

When discussing the psychotic continuum, it is essential to focus on schizotypy and At-Risk Mental States (ARMS). Schizotypy represents the manifest expression of an underlying trait vulnerability for schizophrenia spectrum disorders (15). This construct unfolds along three principal dimensions: the cognitive-perceptual dimension (positive schizotypy: hallucination, delusional phenomena), the interpersonal dimension (social anxiety, constricted affect), and the disorganization dimension (odd behaviors, odd speech) (16, 17). Its expression encompasses a broad range of phenomenology involving personality, subclinical, and clinical psychosis (15).

Individuals with ARMS – also known as at Clinical High Risk (CHR) or Ultra High-Risk (UHR) for psychosis – exhibit a vulnerability of state (i.e., newly emergent attenuated psychotic symptoms, brief and limited psychotic symptoms, reduced social occupational functioning) and or genetic risk based on having a first degree relative with psychosis. These factors increase the likelihood of psychotic onset. The concept of ARMS has settled thanks to UHR criteria for psychosis, one or more of the following conditions should be fulfilled: (a) first-degree relative with a psychotic disorder; (b) diagnosis of schizotypal personality disorder; (c) attenuated/or subthreshold psychotic symptoms, (d) brief limited and intermittent psychotic symptoms (i.e., that have resolved spontaneously within a week of onset) (18). Moreover, individuals with ARMS have consistently low social and occupational functioning or have incurred a decrease in the latter of at least 30% from the previous year (19). The formulation of the At-Risk Mental State (ARMS) construct – alongside the Clinical High Risk (CHR) and Ultra High Risk (UHR) criteria – was undertaken with the objective of identifying individuals who are at increased proximal risk for transitioning into a primary episode of psychosis. Consequently, its significance resides in the early detection and intervention before the onset of full-blown clinical psychosis.

Past research has brought out that people with psychotic disorders, including those with ARMS and schizotypal traits often experience difficulties with mentalization, ToM, and metacognition. According to some authors, excessive focus on self generates dysfunctional metacognitive beliefs (e.g., positive metacognitive beliefs and negative beliefs) that predispose subjects to vulnerability to psychopathology (20, 21). Specifically, positive metacognitive beliefs about psychotic experiences (i.e., belief that worrying/ruminating will help to cope) can lead to hallucinations and delusions, while negative beliefs (i.e., negative beliefs about uncontrollability of thoughts and negative beliefs about thoughts in general) can cause distress (22).

Regarding ToM, the literature presents inconsistent results. On the one hand, deficits in ToM appear to constitute a vulnerability factor for the transition to psychosis in at-risk individuals (23); however, on the other hand, some longitudinal studies conducted on large samples do not support this finding (24).

The existing studies show high heterogeneity in methods, samples, and results. To the best of our knowledge, no recent systematic review that has already been published focuses on this specific topic. This justifies a thorough examination of the nature of the links between mentalizing and psychosis risk aimed at integrating the knowledge accrued in the more recent years.

This systematic review aims to (a) evaluate the links between low mentalizing and both state (ARMS/CHR) and trait (schizotypy) risk for psychosis, (b) compare the difference in mentalizing abilities between individuals with ARMS, schizotypy, full-blown psychosis, and healthy controls. For the purposes of the current review, the construct of mentalizing due to its complex and multifaceted nature will be restricted only to studies that have strictly measured reflective functioning, as well as ToM and metacognition.

## 2. Methods

A systematic review was conducted to identify studies that examined mentalization abilities in individuals with ARMS, schizotypal traits and healthy controls. To ensure a relatively recent comprehensive overview of the literature, the starting year for article publication was set as 2010. The review protocol was developed following the Preferred Reporting Items for Systematic Reviews and Meta-Analysis (PRISMA) guidelines (25) and was registered on PROSPERO (number: CRD42023397594, latest updated on 29/03/2023).

### 2.1. Study selection

In February 2023, data sources for relevant publications on empirical studies were gathered *via* computer-based searches in four databases, namely Google Scholar, Scopus, PubMed, and PsycINFO. Each database was searched independently using three specific iteration research strings: (“At-Risk Mental States”) OR (“Ultra High-Risk”) OR (“Clinical High Risk”) OR (Schizotypy) AND (“Mentalizing”) OR (“Theory of Mind”) OR (“Metacognition”). These strings were selected to encompass a broad range of features related to mentalizing abilities and At-Risk Mental States. Citations were retrieved independently for each iterative search and compiled into a complete list, which was then screened for duplicates and imported into Rayyan for the title and abstract screening. The tool aims to improve the efficiency and transparency of systematic reviews and thanks to the blind review function, it allows the convoluted researchers deputed to evaluate the articles to minimize selection bias. To minimize bias, a third independent judge was included to evaluate articles in which the two main judges did not agree. More details are given in section 2.4.

### 2.2. Inclusion criteria

Articles that present a sample composed of individuals at risk for psychosis by both state (ARMS/CHR) and trait (schizotypy) conditions were included. The ARMS approach to psychosis was introduced in the mid-1990s to describe a state in which there is a heightened risk of developing psychosis (18, 26). To be included in the review, studies were required to:

- have at least one of the following risk conditions: a family history of schizophrenia, schizotypal personality traits, schizotypal personality disorder, the presence of attenuated positive symptoms emerging or worsening, and deterioration of social and occupational functioning. More specifically, we will include the studies considering the following instruments for the assessment of ARMS condition: Comprehensive Assessment of At-Risk Mental States (CAARMS), Structured Interview for Psychosis-risk Syndromes (SIPS), Early Recognition Inventory for the retrospective assessment of the Onset of schizophrenia Checklist (Checklist-ERIRAOS) the companion Scale of Prodromal Symptoms (SOPS), the Basel Screening Instrument for Psychosis (BSIP), the Basic Symptoms (BSABS), and the Schizophrenia Proneness Instrument, Adult Version (SPI-A). All these instruments are usually utilized to assess the ARMS condition. Studies that do not meet the risk criteria and/or have not used a valid assessment including the above-mentioned instruments were automatically excluded;
- investigate the association between psychotic risk and mentalizing abilities;
- evaluate differences in mentalizing abilities (reflective functioning, ToM, and metacognition) between individuals with state or trait risk for psychosis, overt psychosis [according to the Diagnostic and Statistical Manual of Mental Disorders (DSM) or International Classification of Diseases (ICD)], and healthy controls/comparison group;
- be peer-reviewed research articles;
- be original articles. Reviews, meeting abstracts, conference proceedings, notes, letters to the editor, research protocols, patents, editorials, books or chapters, and other editorial materials were deemed ineligible for inclusion in this systematic review;
- be quantitative studies;
- be published between 2010 to June 2023;

### 2.3. Studies inclusion

Two reviewers, F.D.S. and C.R., conducted a thorough review of all non-duplicate titles and abstracts to identify articles that were eligible for inclusion in the study. The same reviewers meticulously analyzed the full text of all pertinent articles and resolved any disagreements by reaching a consensus. In the event of any potential differences in agreement, a third reviewer, O.O., was designated to serve as an arbitrator.

### 2.4. Data extraction

F.D.S. and C.R. independently extracted the following data: type of psychotic risk (CHR/ARMS/UHR and schizotypy), participants, gender, age, methodology involved, type of mentalizing abilities measured instruments and major outcomes.

Data are available in Table 1.

**Table 1:** Studies characteristics according to extraction parameters.

Authors and year	Type of psychotic risk	Gender	Mean age (SD)	Methodology	Construct measured	Instruments	Major outcomes
Barbato et al. (28)	Clinical High Risk (CHR): 153 Help Seeking Control (HSC): 68	M: 88 F: 65	19.7 (±4.2)	Longitudinal	Metacognition	*Metacognition:* Meta-Cognitions Questionnaire (MCQ) *Psychotic risk:* Structured Interview for Prodromal Syndromes (SIPS)	At baseline, the CHR group exhibited significantly higher levels of conviction in negative beliefs related to uncontrollability, thoughts’ uncontrollability, danger, and thoughts in general compared to the help-seeking control (HSC) group.
Boldrini et al. (50)	Ultra-High Risk (UHR): 57 Healthy Control (HC): 53	M: 44 F: 66	16.85 (±2.35)	Longitudinal	Reflective Functioning (RF)	*Mentalization*: Reflective Functioning Scale (RFS) *Psychotic Risk:* Structured Interview for Prodromal Syndromes (SIPS)	There was a negative correlation between mentalization and attenuated psychotic symptoms, additionally, individuals with lower RF were more likely to develop a psychotic disorder.
Brüne et al. (36)	At-Risk Mental States (ARMS): 23 Schizophrenia (SZ): 15 Healthy Control (HC): 21	M: 37 F: 22	24.61 (±4.48)	Cross-sectional	Metacognition	*Metacognition:* Metacognition Questionnaire (MCQ) *Psychotic risk:* Structured Interview for Prodromal Syndromes (SIPS)	Individuals with ARMS displayed higher scores in both “negative beliefs” and “need for control” MCQ subscales, as well as in their overall MCQ scores when compared to the control group. Remarkably, those who later converted to psychosis had higher negative metacognitive beliefs at baseline.
Kong et al. (34)	Ultra-High Risk (UHR): 28 Healthy Control (HC): 28	M: 19 F: 9	20.35 (±3.15)	Cross- sectional	Theory of Mind (ToM)	*ToM:* ToM Picture Stories Task (ToM-PST) *Psychotic risk:* Structured Interview for Prodromal Syndromes (SIPS)	There were no significant differences between the two groups in terms of ToM skills.
Ohmuro et al. (32)	At-Risk Mental States (ARMS): 36 First Episode Psychosis (FEP): 40 Healthy Control (HC): 25	M: 36 F: 65	21.7 (±4)	Experimental	Theory of Mind (ToM)	*ToM*: ToM Picture Stories Task (ToM-PST) *Psychotic risk:* Comprehensive Assessment of At-Risk Mental States (CAARMS)	In ARMS and FEP groups ToM was significantly lower than that of the HC. Differences between ARMS and HC disappeared when controlling for premorbid IQ. ToM deficits in ARMS were confirmed only in the comprehension of higher-order false belief.
Salaminios et al. (30)	Schizotypy: 105	M: 52 F:53	15.72 (±1.91)	Cross- sectional	Reflective Functioning (RF)	*Mentalization:* Reflective Functioning Questionnaire *Schizotypy:* Schizotypal Personality Questionnaire (SPQ)	Schizotypal traits (specifically, social anxiety and odd speech) were associated with RF dysfunctions.
Stanford et al. (23)	Clinical High Risk (CHR): 63 Schizophrenia (SZ): 13 Healthy Control (HC): 24	M: 71 F: 7	24.73 (± 5.83)	Cross- sectional	Theory of Mind (ToM)	ToM: “Apple Task,” “Refrigerator Task,” “The Strange Stories Task” *Psychotic risk:* Structured Interview for Prodromal Syndromes (SIPS)	The higher-order Theory of Mind (ToM) capacity was similarly in CHR and HC. The lowest levels of ToM were obtained from schizophrenic patients. Finally, performance at ToM was influenced by IQ.
Vargas et al. (33)	Clinical High Risk (CHR): 24 Healthy Control (HC): 26	M: 24 F: 23	19.84 (± 2, 47)	Cross-Sectional	Theory of Mind (ToM)	*ToM:* Short Story Task *Psychotic risk:* Structured Interview for Prodromal Syndromes (SIPS)	CHRs did not differ in explicit ToM ability but produced less spontaneous inference of mental states. The negative association between ToM skills and symptoms was confirmed.
Wastler and Lenzenweger (35)	Schizotypy: 40 Negative affect: 30 Healthy control (HC): 46	M: 24 F: 82	19.33 ± (1.73)	Cross-sectional	Theory of Mind (ToM)	*ToM:* Original hinting task and Self-referential hinting task *Schizotypy:* Perceptual Aberration Scale and Magical Ideation Scale	Schizotypal individuals made significantly more hypermentalization errors than both control groups. Moreover, Self-referential hypermentalization was significantly related to referential thinking, aberrant salience, interpersonal schizotypal traits.
Zhang et al. (31)	Clinical High Risk (CHR): 84 Healthy Control (HC): 95 Schizophrenia (SZ): 66	M: 127 F: 118	26.9 (±7.6)	Cross-sectional	Theory of Mind (ToM)	ToM: Reading the Mind in the Eyes Test (RMET) *Psychotic risk:* Structured Interview for Prodromal Syndromes (SIPS)/ Scale of Prodromal Symptoms (SOPS)	CHR and SZ subjects had difficulties in reading the mind. Both CHR and SZ subjects spent almost twice as much time on RMET as HC individuals. For SZ patients a significant positive correlation was found between RMET accuracy and time response.

## 3. Results

**Figure 1:**
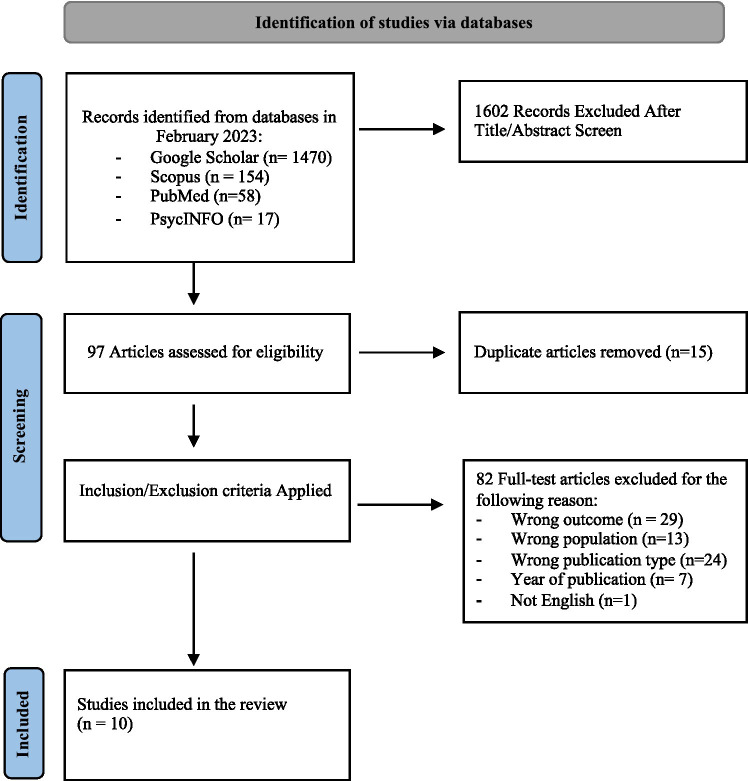
PRISMA flow chart for studies selection.

Of 1,699 studies retrieved from Google Scholar, Scopus, PubMed, and PsycINFO, after screening all non-duplicate titles and abstracts, 1,602 did not fit the preliminary inclusion criteria. Subsequently, the full text of 97 articles was retrieved and the studies were analyzed for the specific inclusion criteria. Of these 97 studies, 87 were excluded. Reasons for exclusion were lack of appropriateness of the study sample (e.g., no state or trait risk for psychosis under previously mentioned criteria were assessed or considered), no pertinence of the construct analyzed in the study (i.e., Reflective functioning, ToM, Metacognition examined), wrong publication type (i.e., review or meta-analysis, qualitative methodology), no English language. Please see Figure 1 for more details about the inclusion/exclusion process. Therefore, 10 articles met the inclusion criteria and were identified as suitable for our review.

In the following paragraphs, study characteristics and results will be presented. Section 3 will focus on specific mentalizing abilities (reflective functioning, ToM, and metacognition) and their association with state and trait risk for psychosis. In addition, the study will present the variations in mentalizing skills among patients with ARMS/CHR, schizotypy, healthy individuals, and patients with full-blown psychosis. Exploring the differences in mentalizing abilities between these groups could provide a better understanding of the progression of psychotic disorders and enable the development of interventions to enhance mentalizing abilities and social functioning.

Detailed information about study characteristics, including the type of risk for psychosis (ARMS, UHR, CHR, schizotypy), participants, gender, age, the methodology involved, type of mentalizing abilities measured (reflective functioning, ToM and metacognition), and major outcomes are presented in Table 1.

### 3.1. Study characteristics

Table 1 displays the study characteristics based on the extraction parameters. The studies included in the review have a sample size dimension that varies from a minimum of 48 (27) to a maximum of 221 participants (28).

Out of the 10 studies extracted, 2 specifically investigate reflective functioning (29, 30), 6 focus on the Theory of Mind (23, 31–35), and 2 on metacognition (28, 36).

Among the 2 studies on reflective functioning, one focuses on the risk for psychosis attributed to schizotypal personality (30), and the other one on the comparison between UHR patients and help seeking controls (29).

Regarding the 6 studies on Theory of Mind, 2 investigate differences between healthy controls and CHR/UHR (33, 34), 3 compare state risk (CHR/UHR/AMRS), people suffering with psychosis, and healthy controls (23, 31, 32), and the last one focuses on the differences between schizotypal, negative affect and healthy control groups (35).

Finally, concerning metacognition, 1 study investigates differences between CHR patients and Help-Seeking Control (28), while the other focuses on differences between ARMS individuals, people suffering with psychosis, and healthy controls (36).

### 3.2. Types of mentalizing ability analyzed (reflective functioning, ToM, metacognition)

#### 3.2.1. Reflective functioning

Two studies have been concerned with investigating the association between risk for psychosis (schizotypy and UHR) and reflective functioning – the operationalization of mentalization (29, 30).

Salaminios et al. (30) aimed to investigate various aspects related to schizotypal personality characteristics – assessed with the Schizotypal Personality Questionnaire (37) and mentalization. Mentalization was measured through the Reflective Functioning Questionnaire (38) a self-report instrument evaluating mentalizing abilities by assessing the degree of certainty and uncertainty with which individuals utilize mental state information to understand their own and others’ behavior. Results revealed that social anxiety and odd speech – features of schizotypy traits – contributed significantly to uncertainty about mental states. These findings highlight schizotypal traits – in particular, social anxiety and odd speech – were associated with RF dysfunctions.

Concerning state risk for psychosis, Boldrini et al. (29) conducted a study that focused on reflective functioning in a clinical sample of Ultra High-Risk (UHR) – status indexed on the Structured Interview for Psychosis-Risk Syndrome (SIPS) (39) – and help seeking controls. The study had multiple objectives, including comparing reflective functioning scores between UHR and help seeking controls, exploring the association between reflective functioning and subclinical psychotic symptoms, and examining the predictive value of reflective functioning for the transition to psychosis in UHR subjects. Reflective functioning was assessed through the Reflective Functioning Scale (40) which provides an index of the ability to mentalize derived from the application of transcripts from the Adult Attachment Interview. It is designed to evaluate whether individuals comprehend attachment-related experiences in terms of mental states. The study found significant differences in mean reflective functioning scores between UHR and help seeking controls, with UHR individuals displaying significantly lower scores. Correlation analysis revealed a negative relationship between reflective functioning and certain attenuated positive psychotic symptoms. Additionally, the analysis confirmed that reflective functioning had a significant effect on the transition to psychosis, explaining over 17% of the variance. Reflective functioning levels emerged as the only dimension capable of predicting the onset of psychosis in this population, with high accuracy in distinguishing UHR individuals who transitioned to psychosis from those who did not develop the disorder. These findings, along with the results from other studies, highlight the presence of mentalizing impairments in both UHR individuals and those with schizotypal traits (29, 30).

#### 3.2.2. Theory of mind (ToM)

Of the 10 selected studies, 5 focused on ToM in individuals at Clinical High Risk (CHR), Ultra High-Risk (UHR) or At Risk Mental State (ARMS) (23, 31–34), and 1 was on differences between schizotypy, negative affect, and healthy control groups in ToM levels (35). Stanford et al. (23) comprehensively evaluated Theory of Mind (ToM) in Clinical High Risk (CHR) individuals – status assessed with SIPS/SOPS (41) – comparing them with healthy controls and people suffering with schizophrenia. ToM was measured by three different tasks: “The Apple Task” (42), “The Refrigerator Task” (43) and “The Strange Stories Task” (44). The “Apple Task” is a cartoon-based assessment that examines whether an individual can determine if an object has been moved in their absence, focusing on first-order false beliefs. Meanwhile, the “Refrigerator Task” is another cartoon-based task that introduces the concept of recognizing deception, emphasizing second-order false beliefs. Finally, the Strange Stories Task is a verbal task that entails advanced inference skills and an understanding of higher-level cognitive processes in others, such as telling white lies, sarcasm, and pretense. No significant differences were found in first-order false belief tasks. Only people suffering with schizophrenia displayed deficits in higher-order ToM tasks. Both CHR patients and the healthy group performed on higher-order tasks similarly but differently from people suffering with schizophrenia – who performed worse than both groups. Notably, none of the ToM measures predicted conversion to psychosis.

Ohmuro et al. (32) compared Theory of Mind (ToM) in healthy controls, First Episode Psychosis (FEP) individuals, and those with ARMS – criteria assessed with Comprehensive Assessment of At Risk Mental States (45). Significant differences were found in mean ToM task scores among the three groups. ToM was assessed with the Theory of Mind Picture Stories Task (46). This assignment consists of six illustrated cartoon narratives portraying instances where two characters collaborate, one character engages in deception towards another, or two characters work together to deceive a third individual. For each narrative, participants were tasked with arranging four cards in a logically sound sequence and responding to inquiries concerning Theory of Mind (ToM) proficiency, such as the deduction of a character’s intent. FEP and ARMS groups differed significantly from healthy controls, with a trend-level difference between FEP and ARMS. The FEP group scored significantly differently from all other groups on second-order false belief tasks, while ARMS individuals showed a trend-level difference.

Two years later, Zhang et al. (31) assessed Theory of Mind (ToM) in Healthy Controls (HC), CHR – condition assessed with SIPS/SOPS (39), and patients suffering with schizophrenia (SZ) to investigate the impact of time consumption on emotion detection. The Reading the Mind in the Eyes Test was administered as a measure of ToM. It consists of the presentation of photographs of the eye region of human faces (42). The results confirmed difficulties in emotional perception for SZ and CHR individuals. Although CHR individuals performed better than the SZ group on ToM tasks, their time consumption was similar. In contrast, the HC group completed the tasks faster with higher accuracy. Additionally, increasing time reaction was associated with improved emotion recognition, highlighting challenges in taking the Reading the Mind in the Eyes Test (RMET).

Vargas et al. (33) compared a CHR group – criteria assessed with SIPS (39) – with a healthy control group to explore correlations between implicit and explicit ToM and positive and negative symptoms. ToM was assessed through Short Story Task (47) which envisages participants read “The End of Something,” a short story by Ernest Hemingway. After reading the story, participants are asked a series of 14 questions to assess comprehension, explicit mental state reasoning and spontaneous mental state inference. In the results, it was found that CHR did not differ to healthy controls in explicit ToM ability, but CHR produced less spontaneous inference of mental states, suggesting impaired implicit and spontaneous ToM ability. From the associations between ToM and symptoms in the CHR, trend-level relationships were found with positive and negative symptoms. This result suggests that CHR individuals exhibit impaired implicit ToM (implying a decreased ability to spontaneously think about the mental states of others), whereas explicit ToM may be relatively more intact at this stage of disease progression (implying that CHR individuals are still able to exercise Theory of Mind and imagine the mental states of others when explicitly elicited).

Kong et al. (34) compared healthy patients with UHR individuals for psychosis – condition assessed with SIPS (39). They investigated impaired ToM skills – evaluated using the ToM Picture Stories Task (46) – and their relationship with schizotypy and executive function in UHR subjects. No significant difference emerged between the groups in ToM skills. Low ToM skills were correlated with positive schizotypy and executive function in UHR individuals.

Wastler and Lenzenweger (35) investigated the relationship between schizotypal traits – assessed with The Schizotypal Personality Questionnaire (48) – and ToM performance. ToM was measured with original and self-referential hinting Task which assess one’s ability to make inferences about self and others’ and mental states based on given indirect speech (43). They expected individuals with high schizotypal traits to perform worse than healthy and psychiatric control groups in overall ToM. Specifically, they anticipated more self-referential hypermentalization errors (i.e., excessive inferences and extrapolations beyond the social cues provided regarding the mental state of others) from the schizotypal group. Notably, the schizotypal group exhibited the highest number of self-referential hypermentalization errors. These errors were correlated with schizotypal trait features, encompassing phenomena such as ideas of reference and anomalous perceptions (indicative of positive schizotypy), alongside manifestations of social anxiety and restricted affect (characteristic of negative schizotypy).

The results obtained from the extracted studies do not confirm the presence of significant differences between CHR/ARMS or UHR and healthy controls for cognitive ToM (23, 32–34). Individuals with CHR, however, take longer (a similar time that is taken by people suffering with psychosis) than healthy controls to attribute emotion to others – a measure of emotional ToM (31). Finally, low ToM abilities have been associated with a wide range of schizotypal manifestations (34); with evidence from one study suggesting that those reporting high schizotypal traits show a tendency towards committing more hypermentalizing errors (35).

#### 3.2.3. Metacognition

Two of the 10 studies identified were concerned with assessing the association between metacognition and CHR/ARMS (28, 36). Barbato et al. (28) conducted a longitudinal study on CHR individuals – condition assessed with SIPS (39), and help-seekers control (HSC). The authors’ objective was to track and analyze metacognitive development over time in a group of CHR. Additionally, they sought to establish whether there was a connection between metacognition and the subsequent onset of psychosis. Metacognition was measured with Meta-Cognitions Questionnaire (MCQ) (20). It has been developed to assess metacognitive beliefs, judgments, and monitoring that are thought to be involved in the development of psychological disturbances. MCQ has five sub-scales: (i) positive beliefs about worry, which includes items related to the idea that worrying is necessary to solve problems; (ii) negative beliefs about uncontrollability of thoughts and corresponding danger, with items related to beliefs about mental and physical danger of worrying and about worrying being uncontrollable; (iii) cognitive confidence, which refers to the efficacy of one’s cognitive skills such as attention and memory; (iv) negative beliefs about thoughts in general, including themes of responsibility, punishment, and superstition whose items concern the negative outcomes that might result from specific thoughts; and (iv) cognitive self-consciousness, which includes items regarding one’s tendency to focus on their own thinking processes (28). From the results emerged that negative beliefs about uncontrollability and cognitive confidence were positively associated with general symptoms in the CHR group. At the baseline, the CHR group reported significantly more conviction in negative beliefs about uncontrollability, negative beliefs about the uncontrollability of thoughts and danger, and negative beliefs about thoughts in general, as well as higher overall MCQ scores compared to the help-seeking controls (HSC), but their conviction in these beliefs decreased over time. Moreover, those who later converted to psychosis had higher negative metacognitive beliefs at baseline. The study suggests impairments in metacognitive beliefs may be linked to the development of genuine psychotic transition. The second selected study was conducted by Brüne et al. (36) and compared a group of ARMS individuals – condition assessed with SIPS/SOPS (39), people suffering with psychosis, and a healthy control with respect to metacognition – assessed with Meta-Cognitions Questionnaire–Revised (49). People with ARMS showed significantly higher scores in both the “negative beliefs” and “need for control” MCQ subscales, as well as overall MCQ, compared to the HSC. Of note, those experiencing psychosis had the highest overall MCQ scores among the groups studied. Both studies identified (28, 36) confirmed that CHR/ARMS individuals present more negative metacognitive beliefs and a higher overall MCQ score compared to respective controls.

## 4. Discussion

To the best of our knowledge, up until the search date of February 2023, this systematic review represents a pioneering endeavor aimed at investigating the role of mentalizing abilities (reflective functioning, ToM, and metacognition) concerning vulnerability to psychosis. The review pursued the following objectives: (a) to assess the associations between low mentalizing and the risk for psychosis due to both state (ARMS/CHR) and trait conditions (schizotypy), and (b) to compare the differences in mentalizing abilities between individuals with ARMS, schizotypy, full-blown psychosis, and healthy controls.

Studies extracted confirmed the negative associations of mentalizing abilities with state (29, 31, 33), and trait (30, 35) risk for psychosis. Specifically, results revealed, that low levels of RF and negative metacognitive beliefs are predictive of transition to psychosis in individuals at risk (28, 29). Negative metacognitive beliefs, such as negative beliefs about the uncontrollability of thoughts and negative beliefs about thoughts in general, appear to be characteristics of individuals who meet the clinical criteria for the ARMS (20).

The research findings regarding differences in mentalizing abilities (23, 28, 29, 32–36) among individuals with ARMS, schizotypy, full-blown psychosis, and healthy controls yielded mixed results. It was observed that negative metacognitive beliefs and the “need for control” tended to be more pronounced in individuals at risk (28, 36). In the context of Theory of Mind (ToM) tasks, some studies failed to identify significant distinctions between CHR patients and healthy controls (23, 33, 34), whereas others reported lower ToM performance levels among schizotypal (35) and CHR individuals (31, 32).

The variability in these findings possibly indicates that in individuals with CHR, their cognitive Theory of Mind (ToM) abilities may remain fairly intact (23, 33, 34). However, they do tend to take significantly more time to recognize emotions in facial expressions compared to healthy controls (31). The need for more time to “read” emotions on the face is plausibly related to the greater difficulty of CHR compared to healthy controls in discerning and understanding the emotional component. Additionally, the discrepancy in the obtained results could be attributed to the effect of cognitive functioning. In fact, studies conducting statistical analyses controlling IQ did not find significant differences between individuals at risk for psychosis and the controls (31, 32), showing that lower IQ scores may be responsible for the differences in ToM performance found between the groups.

To summarize, deficits in reflective functioning and dysfunctional metacognitive beliefs have been identified both in individuals with trait and state risk for psychosis (28–30, 36). The results derived from studies on reflective functioning (29, 30) and metacognitive beliefs (28, 36) align with the perspective that considers impairments in these capacities as potential moderators of the expression of psychotic phenotypes (50–52). Therefore, clinical strategies aimed at enhancing mentalization and metacognition, and thereby promoting resilience, may be more tailored to address the specific needs of individuals across the spectrum of psychosis.

### 4.1. Clinical implications

Mentalization Based Treatment for Psychosis (MBT-P) applied to individuals with both state and trait risk for psychosis may help in the management of attenuated psychotic symptoms. MBT-P, moreover, could be helpful in preventing (53, 54) from the onset of full-blown psychosis through improved long-term social functioning (53, 55). In addition, dysfunctional personality traits are particularly prevalent in this population (56, 57), and an estimated 40% of this population has a personality disorder in comorbidity (50), findings that can justify the application of treatment.

Moreover, the results obtained from the review suggest that the application of strategies to attenuate maladaptive metacognitive beliefs may be equally effective in improving psychotic symptoms in CHR (19, 58–61). Metacognitive training is grounded in theoretical principles that focus on addressing cognitive (e.g., jumping to conclusions) and problem-solving (e.g., poor memory recall) errors and biases, which, in turn, play a significant role in the formation of false beliefs, eventually leading to delusional states (62). Case studies and preliminary trials have shown promising results for metacognitive therapies, showing benefits in improving the sense of self and perceived agency and attenuation in negative metacognitive belief in people suffering with schizophrenia (63, 64). Working on altered mentalization (62) and metacognition (64) should mitigate the phenotypic expression of psychotic disorders, improving the discontinuous experience of the self and psychotic symptomatology in individuals with both state and trait risk for psychosis (65–67).

### 4.2. Limitations and future directions

The present systematic review has several limitations. In general, the research designs used in the selected studies make causal inferences difficult. Therefore, the implementation of more longitudinal studies on the role of mentalization and metacognition and clinical trials applying MBT-P and metacognitive interventions are advocated.

Another constraint to consider is the paucity of research that was both identified and carried out within the temporal scope encompassed by this systematic review. This factor also hampers the ability to make causal inferences.

Finally, only one study (33), among those selected, included neurophysiological or neuroimaging measures in conjunction with psychological/behavioral measures. Considering neuroimaging assessments could further elucidate the links between mentalizing and psychosis risk.

## 5. Conclusion

Considering that impairments in mentalization and metacognition were associated with a wide range of attenuated psychotic symptoms and were predictive of psychotic onset, the assessment of mentalization and metacognition could potentially provide additional prognostic value for individuals along the psychotic continuum (51, 68). In this regard, further research is needed to clarify the relationship between the mentioned dysfunctions and the development and persistence of psychotic and nonpsychotic clinical symptoms. Lastly, it would perhaps be more appropriate to consider good functioning of mentalization and metacognition as protective factors that can improve levels of social and occupational functioning predictive of transition to psychosis (66, 69, 70).

## Data availability statement

The original contributions presented in the study are included in the article/Supplementary material, further inquiries can be directed to the corresponding authors.

## Author contributions

FDS and CR conceived the study protocol. FDS wrote the draft of the manuscript and CR revised it. OO supervised the process and helped create the final manuscript. All authors contributed to the article and approved the submitted version.
