# Supplementary material for: Mentalizing in individuals with state and trait risk for psychosis: a systematic review
Source: Front Psychiatry. 2023 Oct 17;14:1214385. doi: 10.3389/fpsyt.2023.1214385 (PMC10616828; doi:10.3389/fpsyt.2023.1214385)
Supplement: Supplementary file 1 [file Table_1.docx]

Supplementary Material

Mentalizing in Individuals with State and Trait Risk for Psychosis: A Systematic Review

Francesca De Salve^1^*, Chiara Rossi^1^*, Osmano Oasi^1^

^1^ *Department of Psychology, Catholic University of Sacred Heart, Milan, Italy*

*** Correspondence:**

Francesca De Salve

[francesca.desalve@unicatt.it](mailto:francesca.desalve@unicatt.it)

Chiara Rossi

[chiara.rossi1@unicatt.it](mailto:chiara.rossi1@unicatt.it)

**Table 1.** Studies characteristics according to extraction parameters.

| **Authors and year** | **Type of psychotic risk** | **Gender** | **Mean Age (SD)** | **Methodology** | **Construct measured** | **Instruments** | **Major Outcomes** |
| --- | --- | --- | --- | --- | --- | --- | --- |
| (Barbato et al., 2014) | Clinical High Risk (CHR): 153  Help Seeking Control (HSC): 68 | M: 88  F: 65 | 19.7 (±4.2) | Longitudinal | Metacognition | *Metacognition:* Meta-Cognitions Questionnaire (MCQ)  *Psychotic risk:* Structured Interview for Prodromal Syndromes (SIPS) | At baseline, the CHR group exhibited significantly higher levels of conviction in negative beliefs related to uncontrollability, thoughts' uncontrollability, danger, and thoughts in general compared to the help-seeking control (HSC) group. |
| (Boldrini et al., 2020) | Ultra-High Risk (UHR): 57  Healthy Control (HC): 53 | M: 44  F: 66 | 16.85 (±2.35) | Longitudinal | Reflective functioning (RF) | *Mentalization*: Reflective Functioning Scale (RFS)  *Psychotic Risk:* Structured Interview for Prodromal Syndromes (SIPS) | There was a negative correlation between mentalization and attenuated psychotic symptoms, additionally, individuals with lower RF were more likely to develop a psychotic disorder. |
| (Brüne et al., 2019) | At-Risk Mental States (ARMS): 23  Schizophrenia (SZ): 15  Healthy Control (HC):21 | M: 37  F: 22 | 24.61 (±4.48) | Cross-sectional | Metacognition | *Metacognition:* Metacognition Questionnaire (MCQ)  *Psychotic risk:* Structured Interview for Prodromal Syndromes (SIPS) | Individuals with ARMS displayed higher scores in both "negative beliefs" and "need for control" MCQ subscales, as well as in their overall MCQ scores when compared to the control group. Remarkably, those who later converted to psychosis had higher negative metacognitive beliefs at baseline. |
| (Kong et al., 2021) | Ultra-High Risk (UHR): 28  Healthy Control (HC): 28 | M: 19  F: 9 | 20.35 (±3.15) | Cross- sectional | Theory of Mind (ToM) | *ToM:* ToM Picture Stories Task (ToM-PST)  *Psychotic risk:* Structured Interview for Prodromal Syndromes (SIPS) | There were no significant differences between the two groups in terms of ToM skills. |
| (Ohmuro et al., 2016) | At-Risk Mental States (ARMS): 36  First Episode Psychosis (FEP): 40  Healthy Control (HC): 25 | M: 36  F: 65 | 21.7 (±4) | Experimental | Theory of Mind (ToM) | *ToM*: ToM Picture Stories Task (ToM-PST)  *Psychotic risk:* Comprehensive Assessment of At-Risk Mental States (CAARMS) | In ARMS and FEP groups ToM was significantly lower than that of the HC. Differences between ARMS and HC disappeared when controlling for premorbid IQ.  ToM deficits in ARMS were confirmed only in the comprehension of higher-order false belief. |
| (Salaminios et al., 2021) | Schizotypy: 105 | M: 52  F:53 | 15.72 (±1.91) | Cross- sectional | Reflective functioning (RF) | *Mentalization:* Reflective Functioning Questionnaire  *Schizotypy:* Schizotypal Personality Questionnaire (SPQ) | Schizotypal traits (specifically, social anxiety and odd speech) were associated with RF dysfunctions. |
| (Stanford et al., 2011) | Clinical High Risk (CHR): 63  Schizophrenia (SZ): 13  Healthy Control (HC): 24 | M: 71  F: 7 | 24.73 (± 5.83) | Cross- sectional | Theory of Mind (ToM) | ToM: “Apple Task”, “Refrigerator Task”, “The Strange Stories Task”  *Psychotic risk:* Structured Interview for Prodromal Syndromes (SIPS) | The higher-order Theory of Mind (ToM) capacity was similarly in CHR and HC. The lowest levels of ToM were obtained from schizophrenic patients. Finally, performance at ToM was influenced by IQ. |
| (Vargas et al., 2019) | Clinical High Risk (CHR): 24  Healthy Control (HC): 26 | M: 24  F: 23 | 19.84 (± 2, 47) | Cross-Sectional | Theory of Mind (ToM) | *ToM:* Short Story Task  *Psychotic risk:* Structured Interview for Prodromal Syndromes (SIPS) | CHRs did not differ in explicit ToM ability but produced less spontaneous inference of mental states. The negative association between ToM skills and symptoms was confirmed. |
| (Wastler and Lenzenweger, 2019) | Schizotypy: 40  Negative affect: 30  Healthy control (HC): 46 | M: 24  F: 82 | 19.33 ± (1.73) | Cross-sectional | Theory of Mind (ToM) | *ToM:* Original hinting task and Self-referential hinting task  *Schizotypy:* Perceptual Aberration Scale and Magical Ideation Scale | Schizotypal individuals made significantly more hypermentalization errors than both control groups. Moreover, Self-referential hypermentalization was significantly related to referential thinking, aberrant salience, interpersonal schizotypal traits. |
| (Zhang et al., 2018) | Clinical High Risk (CHR): 84  Healthy Control (HC): 95  Schizophrenia (SZ): 66 | M: 127  F: 118 | 26.9 (±7.6) | Cross-sectional | Theory of Mind (ToM) | ToM: Reading the Mind in the Eyes Test (RMET)  *Psychotic risk:* Structured Interview for Prodromal Syndromes (SIPS)/ Scale of Prodromal Symptoms (SOPS) | CHR and SZ subjects had difficulties in reading the mind. Both CHR and SZ subjects spent almost twice as much time on RMET as HC individuals. For SZ patients a significant positive correlation was found between RMET accuracy and time response. |
